# Diagnosis and invasive staging: Non-surgical invasive mediastinal staging. Endobronchial ultrasound

**Published:** 2020-09-02

**Authors:** Virginia Pajares, Alfons Torrego, Elisabeth Martínnez-Téllez, Juan Carlos Trujillo-Reyes

**Affiliations:** ^1^Respiratory Medicine; ^2^Biomedical Research Institute Sant Pau (IIB-Sant Pau), Barcelona, Spain; ^3^Department of Thoracic Surgery, Hospital de la Santa Creu i Sant Pau

**Keywords:** Endobronchial ultrasound, Endobronchial ultrasound-transbronchial needle aspiration, Lung cancer, Lung cancer staging

## Abstract

The diagnosis and staging of lung cancer are an important process that identifies treatment options and guides disease prognosis. Therefore, an accurate mediastinal lymph node (LN) staging is required not only to offer the appropriate treatment but also to avoid unnecessary invasive procedures. At present, endobronchial ultrasound (EBUS)-transbronchial needle aspiration is the preferred modality for sampling mediastinal lymph nodes because of its minimally invasive nature and high diagnostic yield. In this review, we discuss the utility of EBUS in mediastinal LN staging of non-small cell lung cancer (NSCLC).

**Relevance for patients:**

The use of EBUS-TBNA in the diagnosis of mediastinal and hilar LN pathology has become in an essential endoscopic technique and the first step for staging of lung cancer.

## 1. Introduction

Endobronchial ultrasound (EBUS) is a minimally invasive technique used to diagnose mediastinal and pulmonary tumors and nodes, that, in the last decade, have become a fundamental tool in diagnosing and staging lung cancer, a field in which most research to date has focused [1-5]. Two approaches to EBUS, which guides fine-needle aspiration (FNA) of mediastinal and hilar adenopathies and tumors adjacent to the airway, are available: Radial probe EBUS (RP-EBUS), which directs the puncture without real-time guidance, and linear EBUS transbronchial needle aspiration (TBNA), in which the needle is guided by ultrasound (US).

## 2. Radial Probe-Endobrobronchial Ultrasound (RP-EBUS)

RP-EBUS, the first endobronchial US technique to become available, was used in the early 1990s to perform mediastinal staging [2,6]. It consists of a US mini-wave, which, when introduced through a conventional bronchoscope working channel, allows 360° visualization and viewing of the tracheobronchial wall structure, with its different layers, and the mediastinal nodes. At the distal end of the probe a small transducer rotates through a mechanical motor unit and provides images of cross-sections of the mediastinal structures. The use of high-frequency US (20 MHz) ensures a good image resolution at the expense of depth. This technique is currently mainly used to diagnose peripheral pulmonary nodules and is not indicated for mediastinal staging.

## 3. Real-time Endobronchial Ultrasound-guided Transbronchial Needle Aspiration (EBUS –TBNA)

In 2002, a flexible US bronchoscope was developed with a convex transducer at its distal end for real-time FNA. The use of the convex probe EBUS to perform TBNA under direct US guidance was first reported in preliminary studies [1]. The utility of EBUS-TBNA in the evaluation of hilar and mediastinal lymph nodes in patients with non-small cell lung cancer (NSCLC) was confirmed in multiple studies. EBUS-TBNA can be used to simultaneously diagnose, stage, and obtain cellular material for ancillary tests, including molecular analysis for prognosis and targeted therapy [7-14]. Numerous studies have demonstrated that EBUS-TBNA is an accurate, minimally invasive, and cost-effective procedure for the staging of mediastinal lymph nodes when compared with other methods, including mediastinoscopy [15-18].

### 3.1. Lung cancer diagnosis and staging indications

The main indication of EBUS-TBNA is mediastinal staging in patients with NSCLC, with clinical practice guidelines on mediastinal diagnosis and staging considering EBUS-TBNA to be a key tool [19-23]. The goal is to evaluate possible mediastinal lymph node (LN) involvement, provided there is no evidence of distant metastasis. This kind of staging is useful to determine prognosis and decide a treatment plan. EBUS-TBNA allows the needle to be viewed in real time. Linear EBUS, which allows the 2, 3p, 4, and 7 mediastinal and 10 and 11 hilar stations to be explored, results in a high yield, even for nodes with smaller axes measuring 5-10 mm [24,25]. The results of published meta-analyses confirm this high diagnostic yield [19,26-30]. Rapid on-site evaluation (ROSE) by an expert pathologist significantly increases yield by reducing the number of non-representative samples. Studies have shown that ROSE improves the sample adequacy rate and diagnostic yield. Davenport *et al*. [31] demonstrated that ROSE produced a significant increase in the percentage of specimens containing malignant cells, from 31% to 56%, and a large decrease in the percentage of specimens that were inadequate for diagnosis, from 56% to 18%. The utility of ROSE has shown reduction in the number of needle passes and the sites biopsied because it may not be necessary to biopsy LN if a higher-stage LN is positive for malignant cells by on-site evaluation [7,32]. In the absence of ROSE, diagnostic performance is based on the number of punctures, which range from a single puncture to three punctures of the same LN in 69.8% and 95.3% of cases, respectively [33]. For a meta-analysis of 11 studies (1299 patients) of NSCLC staging using EBUS [27], sensitivity was 93% and specificity was 100%. In another study, a subgroup analysis highlighted that using chest computed tomography (CT) or positron emission tomography (PET) to select patients with abnormal lymph nodes and the availability of immediate cytopathological diagnoses were factors that independently increased overall sensitivity and specificity to 94% and 97%, respectively [20]. Other studies indicate that assessing US characteristics during the examination yields relevant predictive information on malignancy or benignity, for example, diameter, spherical or ovoid shape, heterogeneous or homogeneous echogenicity, central cavitation, and circulation inside the LN [34,35].

Combining EBUS-TBNA with other endoscopy techniques such as endoscopic US (EUS) means that the mediastinal study can include exploration of stations that cannot be explored using EBUS. While EUS emerged in the diagnosis and staging of digestive neoplasms, it can also be used, combined with EBUS, for NSCLC diagnosis and mediastinal staging, as well as for the evaluation of certain distant metastases. EUS allows the posteroinferior mediastinum and the 4L, 5, 7, 8, and 9 stations to be analyzed. In a systematic review of 18 studies published in 2007, sensitivity and specificity values for FNA using EUS to detect malignant mediastinal adenopathies were 83% and 97%, respectively [36]. EUS-FNA can also detect subdiaphragmatic metastases (left adrenal gland, coeliac trunk, and liver lymph nodes) [37] as well as mediastinal invasion (T4) [38].

It is currently unclear whether EBUS and EUS combined should be used systematically in all patients and in all regions accessible to those procedures or whether it should be used exclusively for cases with inaccessible or difficult-to-reach adenopathies [15,39-41].

### 3.2. NSCLC restaging indications

The usefulness of EBUS-TBNA for mediastinal restaging has not been established. Although mediastinoscopy is the gold standard, its repetition is very complex because of possible adhesions and fibrosis. So far, studies published on EBUS-TBNA include one on 363 patients with histological Stages IIIA [42-46]; the prospective study by Herth *et al*. [42] included 124 patients with mediastinal LN disease (IIIA) undergoing induction chemotherapy, for whom chest CT, EBUS, and thoracotomy with lymphadenectomy were performed. The chest CT showed stability in 46.7% of patients, EBUS confirmed persistent nodal metastasis in 72% of patients and lymphadenectomy showed disease persistence in 94% of patients. Other studies that analyzed mediastinal restaging after induction treatment point to highly variable mediastinal LN involvement prevalence rates of between 20% and 88%. In general, in the initial staging of NSCLC, EBUS-TBNA results in the lower sensitivity and similar specificity [47]

## 4. Mediastinal Staging Algorithm

Clinical practice guidelines have proposed different mediastinal staging algorithms [19-21]. With all of them coinciding in including EBUS-TBNA as the first-line technique for confirming NSCLC mediastinal involvement, since yield is comparable to that of mediastinoscopy when combined with EUS [48].

However, the algorithms differ in EBUS-TBNA indications for patients with normal mediastinum images and the confirmation of negative results obtained using endoscopic methods [49-51]. The first multicenter randomized trial that compared surgical and endoscopic mediastinal staging methods with imaging methods (diameter >10 mm in chest CT or positive PET) was the ASTER study [15] of patients with NSCLC and mediastinal adenopathies with central tumors or suspected N1 involvement; the authors concluded that sensitivity for nodal metastasis diagnosis was 79% for the single surgical staging method and 94% for EUS, followed by surgery if the EUS examination was negative. However, Tournoy *et al*. [52] showed that, in terms of probabilities of detecting malignant adenopathies, when the mediastinal image was normal, the probability was the same (5%) for EUS alone and for EUS followed by surgical staging; however, when the mediastinum image was pathological, the probability for EUS followed by surgical staging was higher (20%); this would suggest that only negative EBUS results for pathological mediastinal images should be confirmed surgically. However, this issue remains open to debate and there is no consensus in the guidelines regarding the need for surgical confirmation of negative EBUS in patients with normal mediastinal images.

Ong *et al*. [53] reported a finding similar to that obtained in a previous prospective study [54], namely, that, in patients with normal mediastinal images, LN metastases, were significantly related to centrally located tumors, 67% of which were located in the upper lobes. Similarly, for a large sample, Yazdi *et al*. [55] found that centrally located tumors, along with positive PET, were false negative predictors for patients with negative EBUS-TBNA.

Different studies have shown that even when chest CT or PET scans indicate alterations, the reliability of negative EBUS-TBNA results varies greatly depending on the characteristics of the neoplasm, the adenopathies (location, US features, size, tracer uptake in PET), the procedure, endoscopist, and pathologist experience and the sample quality [47,56,57].

The key issues currently seem to be the correct choice of the sequence of examinations and the need for confirmation of negative results obtained by EBUS. In general, if puncture techniques are negative, surgical confirmation is recommended in cases of a high post-test malignancy probability.

**Figure 1:**
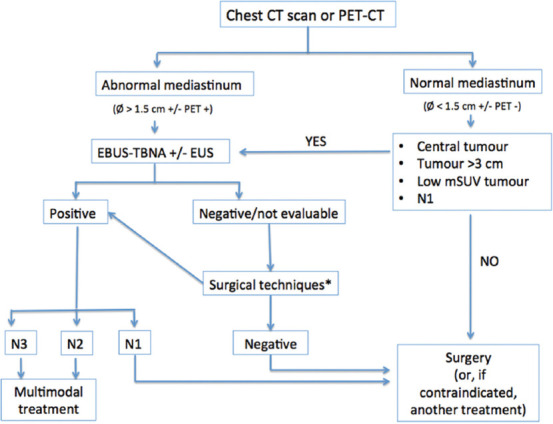
Proposed mediastinal staging algorithm. Modified from Sánchez de Cos J et al. [6]. *Surgical techniques: mediastinoscopy, mediastinotomy, extended cervical mediastinoscopy, thoracoscopy, transcervical extended mediastinal lymphadenectomy, and video-assisted mediastinoscopic lymphadenectomy.

As a diagnostic algorithm (Figure 1), according to the latest Spanish Society of Pulmonology and Thoracic Surgery (SEPAR) [20] guidelines on lung cancer staging to evaluate the mediastinum and to detect possible distant metastasis, PET-CT is indicated for patients with Stage IA-IIIA who are potential candidates for radical treatment. In patients with suspected pathologic LN involvement according to imaging techniques, cytohistological confirmation should be obtained by invasive techniques. If EBUS-TBNA results are negative, this should be confirmed using surgical techniques, usually mediastinoscopy.

If PET-CT results are negative a cytohistological study of the mediastinum should be performed using endoscopic or surgical techniques in the following circumstances: Primary tumor >3 cm, mainly with a very high standardized uptake value (SUV); mediastinal adenopathies in the chest CT scan (diameter >1.5 cm); a central tumor in contact with the mediastinum, primary tumor with a low maximum SUV; or suspected N1 involvement according to CT or PET-CT [25,33].

### 4.1. Mediastinal staging strategy

Although there is no consensus on what the standard for a EUS examination should be, the following procedure is recommended [20]:

- Explore and puncture all suspicious nodes according to the PET-CT, sequentially discarding N3, N2, and N1.
- Explore all the N3 LN stations with the intention of a radical cure and puncture lymph nodes ≥5 mm in diameter.

Representative samples can be obtained (i.e., cytological or evaluable and negative diagnoses of malignancy) from the 4R, 4L, and 7 regions in over 80% of patients staged with EBUS-TBNA, when the negative predictive value is 93.6% [58].

**Figure 2:**
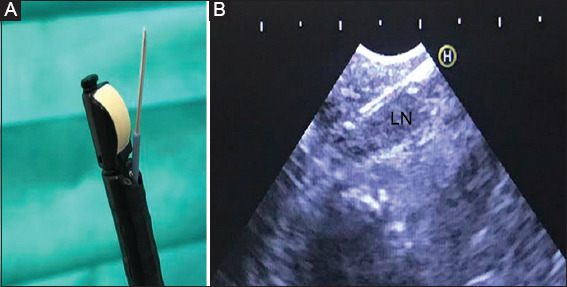
(A) Convex transducer of endobronchial ultrasound and transbronchial needle aspiration in the work channel. (B) Real-time of endobronchial ultrasound and the needle puncture of lymph node.

## 5. EBUS-TBNA Complications

EBUS-TBNA is considered a safe technique and is generally well tolerated by patients. Its contraindications are few and are similar to those for conventional bronchoscopy (unstable ischemic heart disease, arrhythmias and severe hypoxia). Possible problems for anticoagulated or antiplatelet patients should be corrected by withdrawing antiaggregant medication 5-7 days before examination. Eapen *et al*. [59], in a prospective study in 1317 patients of enhanced use of EBUS and other endoscopic techniques, reported an incidence of 1.44% of serious complications, most frequently, pneumothorax, and respiratory failure. The authors recorded one death although note that other endoscopic techniques were also used in that study (transbronchial biopsy). In a systemic review of 190 studies, Von Bartheld *et al*. [60] reported a complications rate of just 0.14%, indicating infection (0.02%), and pneumothorax (0.02%) as the most frequent adverse events and reporting no deaths.

## 6. Conclusion

EBUS-TBNA is a safe and minimally invasive technique key to the diagnosis and mediastinal staging of patients with suspected or confirmed lung cancer. However, when results are negative, further studies are necessary to ensure correct diagnoses.

### Conflicts of Interest Statement

The authors have no conflicts of interest to declare.

### Funding

The authors declare that the manuscript was conducted in the absence of any commercial or financial relationships.
